# Screening and Brief Intervention in Primary Care Settings

**Published:** 2004

**Authors:** Michael F. Fleming

**Affiliations:** Michael F. Fleming, M.D., M.P.H., is a professor of family medicine at the University of Wisconsin Medical School, Madison, Wisconsin

**Keywords:** identification and screening for AOD (alcohol and other drug) use, Alcohol Use Disorders Identification Test, health risk assessment, binge drinking, patient interview, primary health care, general practitioner, brief intervention, prevention, counseling

## Abstract

Primary care practitioners are in a unique position to identify patients with potential alcohol problems and intervene when appropriate. Screening, the process by which practitioners can identify at-risk drinkers, can be followed by one-time or repeated short counseling sessions, known as brief interventions, which are designed to help the patient reduce drinking and minimize related problems. Varied levels of screening and brief intervention can be implemented in the primary care setting, depending on patient and physician factors. Although screening and brief intervention are valuable tools, they are underutilized in primary care practices. Strategies that may help increase physicians’ use of these techniques in the primary care setting include skills-based role-playing, performance feedback, clinical protocols, clinic-based education, and training by credible experts.

Health care practitioners who work in primary care settings have the important responsibility of overseeing their patients’ general health and welfare. In this role, they must be vigilant in identifying a host of potential health problems. Because many health problems can result from the misuse of alcohol, primary care practitioners can help patients avoid these problems by recognizing problematic alcohol use early. According to the National Institute on Alcohol Abuse and Alcoholism (NIAAA), men may be at risk for alcohol-related problems if their alcohol consumption exceeds 14 standard drinks^1^ per week or 4 drinks per day, and women may be at risk if they have more than 7 standard drinks per week or 3 drinks per day (NIAAA 2003). In one study, about 20 percent of primary care patients reported levels of consumption that exceeded these guidelines (Fleming et al. 1998). In addition, 35 percent of the men and 16 percent of the women participating in the study reported binge drinking (i.e., consuming six or more drinks per occasion^2^) during the 90 days before the survey. Other studies of primary care patients have estimated rates of alcohol abuse or dependence at 2 percent to 9 percent of study participants (Reid et al. 1999).

Primary care clinicians are in a unique position to recognize patients with potential alcohol problems and intervene when appropriate. Screening, an interview process by which practitioners can identify at-risk drinkers, can be followed by one-time or repeated short counseling sessions, known as brief intervention, which are designed to help the patient reduce drinking and minimize related problems. This article will examine how screening and brief intervention can be implemented in the primary care setting. The levels of screening and intervention described here are summarized in the accompanying table.

## Screening for At-Risk Drinking and Alcohol Abuse and Dependence in Primary Care Settings

Screening in primary care can vary in scope and intensity from only one question to an extensive assessment using a standardized questionnaire. The level of screening a clinician uses can depend on the patient population, whether patients have co-occurring medical or psychiatric problems, physician skills and interest, and the amount of time available. To make the most of the opportunity to reduce or alleviate patients’ alcohol problems, it is critical that physicians practice some level of screening with all patients.

**Table:** Levels of Screening and Brief Intervention

Screening Level	When to Use This Level	Screening Technique
1	If only one question is possible	On any single occasion during the past 3 months, have you had more than 5 drinks containing alcohol? (Taj et al. 1998).
2	With all patients who report drinking alcohol, if time allows, or for patients who respond “yes” to a level 1 screening question	On average, how many days per week do you drink alcohol? On a typical day when you drink, how many drinks do you have? What is the maximum number of drinks you had on any given day in the past month? (NIAAA 1995, 2003).
3	If level 2 screening reveals that the patient may be at risk for alcohol-related problems (i.e., for men whose alcohol consumption exceeds 14 standard drinks per week or 4 drinks per day, or for women whose consumption exceeds 7 standard drinks per week or 3 drinks per day), or if the clinician suspects that the patient is minimizing his or her alcohol use	The 10-question Alcohol Use Disorders Identification Test (AUDIT) (Saunders et al. 1993).

**Brief Intervention Level**	**When to Use This Level**	**Brief Intervention Technique**

1	If screening results determine that intervention is necessary but time is limited	Simply state concern that the patient’s drinking exceeds recommended limits and could lead to alcohol-related problems. Recommend that the patient minimize or stop drinking (WHOBISG 1996).
2	If referral to a specialist is not necessary; if abstinence is not necessarily the goal	Project TrEAT (Trial for Early Alcohol Treatment) protocol: two brief face-to-face sessions scheduled 1 month apart, with a followup telephone call 2 weeks after each session (Fleming et al. 2002).
3	If the patient has symptoms of alcohol abuse or dependence; if abstinence is the primary goal	Motivational enhancement, referral to a specialist.

### Level 1 Screening

Clinicians under strict time constraints may have only enough time to ask a patient one screening question about alcohol consumption. One study (Taj et al. 1998) has shown that a positive response to the question “On any single occasion during the past 3 months, have you had more than 5 drinks containing alcohol?” accurately identifies patients who meet NIAAA criteria for at-risk drinking and those who meet the criteria for alcohol abuse and dependence specified in the *Diagnostic and Statistical Manual of Mental Disorders, Fourth Edition* (APA 1994).

### Level 2 Screening

For clinicians who have time for more than one question, a series of questions recommended by NIAAA (2003) can reveal the patient’s frequency and level of alcohol use. These questions should be asked of all patients on an annual basis or in response to problems that may be alcohol related. They could be included in a pre-exam interview conducted as part of the patient’s check-in process.

- For all patients: Do you drink alcohol, including beer, wine, or distilled spirits?
- For current drinkers:
  – On average, how many days per week do you drink alcohol?
  – On a typical day when you drink, how many drinks do you have?
  – What is the maximum number of drinks you had on any given day in the past month? (NIAAA 1995, 2003).

Patients who report binge drinking, male patients who report drinking more than 14 drinks per week, and female patients who have more than 7 drinks per week should receive brief intervention.

### Level 3 Screening

If level 2 screening reveals that the patient may be at risk for alcohol-related problems, or if the clinician suspects that the patient is minimizing his or her alcohol use, the clinician may proceed to additional qualitative questions, which can reveal more information about the nature and extent of the problem. For example, the 10-question Alcohol Use Disorders Identification Test (AUDIT) (Saunders et al. 1993) includes questions about the quantity and frequency of alcohol use, as well as binge drinking, dependence symptoms, and alcohol-related problems. It is more accurate than other screening methods in identifying at-risk drinking (Fiellin et al. 2000). Research has supported the accuracy of the AUDIT when used with women and minorities (Reinert and Allen 2002). This screening tool also has had promising results when tested with adolescents and young adults; it is less accurate with older patients, although further research is needed in these populations (Reinert and Allen 2002; Chung et al. 2000).

**Screening and Intervention for Alcohol Misuse: Recommendations of the U.S. Preventive Services Task Force (USPSTF)**

The U.S. Preventive Services Task Force (USPSTF) is a 20-member nongovernmental panel commissioned by the U.S. Public Health Service whose mission is to systematically review the scientific evidence on individual clinical preventive services and to recommend the services practitioners should routinely offer. (See the sidebar by Russell in the companion issue of *Alcohol Research & Health*, “Screening and Brief Intervention, Part I: An Overview,” for more information about the USPSTF and its review process.) In 2004, the USPSTF released a recommendation that primary care settings are suitable locations for offering screening and behavioral interventions to reduce alcohol misuse by adults, including pregnant women, as follows:

The USPSTF found good evidence that screening in primary care settings can accurately identify patients whose levels or patterns of alcohol consumption do not meet criteria for alcohol dependence, but place them at risk for increased morbidity and mortality, and good evidence that brief behavioral counseling interventions with followup produce small to moderate reductions in alcohol consumption that are sustained over 6- to 12-month periods or longer. The USPSTF found some evidence that interventions lead to positive health outcomes 4 or more years post-intervention, but found limited evidence that screening and behavioral counseling reduce alcohol-related morbidity. The evidence on the effectiveness of counseling to reduce alcohol consumption during pregnancy is limited; however, studies in the general adult population show that behavioral counseling interventions are effective among women of childbearing age. The USPSTF concluded that the benefits of behavioral counseling interventions to reduce alcohol misuse by adults outweigh any potential harms.

SOURCE: U.S. Preventive Services Task Force. “Screening for Alcohol Misuse.” Available at: www.preventiveservices.ahrq.gov. April 2004.

Computerized versions of the AUDIT or other instruments can be used in conjunction with other health assessment questionnaires.

## Brief Intervention in Primary Care

Brief intervention in primary care, like screening, can be simple and short or more extensive, possibly including referral to a substance abuse specialist. The level of intervention needed for a particular patient depends on the severity of the patient’s alcohol abuse or dependence, whether the patient also uses tobacco or illicit drugs or has co-occurring medical or psychiatric conditions, as well as on the clinical setting, the clinician’s skills and level of interest, and the time available. Clinicians with limited time may want to use a level 1 intervention for all patients who use alcohol above recommended limits and refer those patients who do not respond to a level 1 intervention to an alcohol treatment specialist at the followup visit.

### Level 1 Brief Intervention

The most basic level of brief intervention consists of a simple statement or two. This level is strictly physician centered. The clinician states simply that he or she is concerned about the patient’s drinking, that it exceeds recommended limits and could lead to alcohol-related problems. The clinician also makes a recommendation that the patient minimize or stop drinking (WHOBISG 1996).

### Level 2 Brief Intervention

This level of brief intervention involves two brief face-to-face sessions scheduled 1 month apart, with a followup telephone call 2 weeks after each session. This intervention was studied in Project TrEAT (Trial for Early Alcohol Treatment), a large-scale clinical trial conducted in primary care practices, and found to be effective up to 4 years later (Fleming et al. 2002). Patients in the intervention group reported reduced alcohol use, fewer days of hospitalization, and fewer emergency department visits compared with control group patients. This intervention may be especially useful with patients who are experiencing alcohol-related harm but who do not necessarily need referral to a specialist and may not need to stop drinking completely.

### Level 3 Brief Intervention

A more extensive level of brief intervention that takes 15 to 20 minutes, a level 3 intervention can be administered by a primary care clinician or an office-based therapist. It may involve the use of strategies to increase a patient’s motivation to change his or her alcohol use, such as providing feedback about the negative consequences of the patient’s drinking and the risks of further problems, as well as information about the potential benefits of abstinence. This type of intervention often is used with patients who have symptoms of alcohol abuse or dependence, for whom abstinence may be the primary goal. Referral to a specialist is often a component of this type of intervention.

## Research on the Effectiveness of Brief Intervention

Research has established the effectiveness of brief intervention in decreasing alcohol consumption among both male and female primary care patients, and among older and younger adults (Whitlock et al. 2004). Interventions that involve repeated contact generally are more effective than single-contact interventions (Whitlock et al. 2004). A review of studies reported that intervention participants reduced their alcohol consumption an average of 13 percent to 34 percent compared with the control group (USPSTF 2004). In addition, a recent meta-analysis concluded that brief interventions can reduce mortality rates among problem drinkers by an estimated 23 to 26 percent (Cuijpers et al. 2004). Most studies of brief intervention have been conducted in primary care practices, thus establishing that tightly controlled clinical settings are not necessary to show the positive results of this type of intervention.

## Putting Research Into Practice

Screening and brief intervention are underutilized in primary care practices. One survey of primary care physicians found that although most (88 percent) reported asking their patients about alcohol use, only 13 percent used standard screening instruments (Friedmann et al. 2000). In a survey of primary care patients, more than 50 percent said their primary care physician did nothing about their substance abuse; 43 percent said their physician never diagnosed it (National Center on Addiction and Substance Abuse [CASA] 2000).

Research suggests that routine educational methods such as lectures and handouts have limited effectiveness in changing physicians’ approaches (Davis et al. 1995). To increase physicians’ use of screening and brief intervention in the primary care setting, other strategies are needed. Effective group education strategies include the use of skills-based role-playing, performance feedback, clinical protocols, clinic-based education, and training by credible experts (Davis et al. 1995).

### Role-Playing

Role-playing can be an especially useful tool for helping physicians become more comfortable with alcohol screening questions and interview techniques, because it allows them to rehearse their skills before they interact with patients (Fleming 1997). One study of a brief intervention skills training program reported that a 90-minute training workshop followed by a 30-minute, one-on-one feedback session 2 to 6 weeks later significantly changed clinicians’ attitudes and increased their skills and knowledge (Ockene et al. 1997).

### Performance Feedback

Giving health care providers feedback about their practice performance and patient outcomes compared with the performance of other providers (Greco and Eisenberg 1993) can be used to introduce a new procedure, or it can be part of a clinic’s quality assurance system. Examples of effective feedback include confidential performance evaluations based on medical record reviews, written reports from quality assurance committees, and information obtained from patient satisfaction questionnaires. Peer-review feedback is increasingly used by managed care organizations to modify physician behavior, especially in the prevention field. Peer-review feedback information also is used to monitor the quality of care that patients receive and can serve as the basis for financial incentives for physicians (Fleming 1997). Feedback is most effective in changing behavior when it is delivered in a timely fashion, includes comparisons with peers, and is combined with education and either incentives or administrative changes (Schwartz and Cohen 1990).

### Clinic-Based Approach

Clinic-based systems use a comprehensive approach to incorporate new clinical activities into routine care. All members of the clinic staff participate in this type of system, which may use written or computerized screening, or include screening questions as part of a general health interview. In addition, a reminder system can be established to prompt clinicians to ask alcohol screening questions. Literature such as alcohol information booklets, information about self-help group meetings, and referral information also can be provided (USDHHS 2000; Fleming and Graham 2001).

### Clinic-Based Education

Educational activities conducted in the clinical setting can include presentations to physicians, skills training through role-playing, performance feedback, or discussions on pertinent topics (e.g., how to overcome staff resistance to incorporating new procedures). One study compared the effects of face-to-face outreach visits by clinical pharmacists with distributing written materials on changing physicians’ prescribing patterns (Soumerai and Avorn 1990). Educational visits significantly changed the physicians’ prescribing patterns, and the strength of the effect depended on the number of one-on-one followup visits by the clinical pharmacist: the more visits, the greater the change in prescribing patterns. This study concluded that brevity, repetition, and reinforcement of recommended practices are important elements in changing physician behavior (Soumerai and Avorn 1990).

### Expert Educators

The use of credible experts as educators is particularly valuable in the alcohol field, in which societal and health care system barriers may impede the incorporation of alcohol screening into routine clinical care. Respected colleagues can help overcome these barriers by legitimizing and providing the scientific rationale for implementing alcohol screening procedures (Fleming 1997).

**At a Glance**

**Screening and Brief Intervention for Alcohol Use Problems in Primary Care**

According to two surveys of physicians and patients, regarding alcohol screening:

- 94 percent of primary care physicians missed or misdiagnosed alcohol-abusing patients (that is, when presented with early symptoms of alcohol abuse in adult patients, the physicians failed to include substance abuse among the five diagnoses they offered).^b^
- 88 percent of physicians said they asked new outpatients whether they drank alcohol, but only 13 percent used a formal alcohol screening tool.^a^
- 19.9 percent of primary care physicians considered themselves “very prepared” to identify alcoholism.^b^
- 54.8 percent of patients believed that physicians do not know how to detect addictions.^b^

and regarding brief intervention:

- The majority of physicians said they usually or always recommend 12-step groups to problem-drinking patients.^a^
- 53.7 percent of patients said their primary care physician did nothing about their substance abuse, 43 percent said their physician never diagnosed it, and 10.7 percent believed their physician knew about their addiction but did nothing about it.^b^
- 74.1 percent of patients said their primary care physician was not involved in their decision to seek treatment, and 16.7 percent said the physician was involved “a little.”^b^

SOURCES: ^a^Friedmann et al. 2000; ^b^National Center on Addiction and Substance Abuse 2000.

## Conclusion

Primary care physicians can play a valuable role in identifying and helping patients who use alcohol above recommended limits or who have symptoms of alcohol abuse or dependence. The screening methods discussed in this report will identify most patients seen in clinical settings who need to reduce or discontinue their alcohol use. The reliability and validity of these methods are similar to those of screening techniques used to detect chronic illnesses such as hypertension, diabetes, and lipid disorders. Brief intervention has been shown to be effective in reducing alcohol use and long-term alcohol-related harm.

The majority of patients seen in clinical settings, however, are not asked about alcohol use, and those who drink above recommended limits often do not receive brief intervention treatment. To reduce the frequency of alcohol-related harm among their patients, it is critical for physicians and other health care providers to routinely practice some type of screening and brief intervention. Health care organizations can use effective educational tools and programs to help primary care clinicians integrate screening and brief intervention into their practice.

## Footnotes

1 A standard drink is defined as one 12-ounce bottle of beer, one 5-ounce glass of wine, or 1.5 ounces of distilled spirits.

2 This definition of binge drinking differs from that established by the National Advisory Council of the National Institute on Alcohol Abuse and Alcoholism, which defines binge drinking as consuming four or more drinks for women, or five or more drinks for men, in about 2 hours.
